# Combined Endocyclophotocoagulation and Phacoemulsification in Patients with Glaucoma of African Descent

**Published:** 2019-10-01

**Authors:** Corey W. Waldman, Manishi Desai, Effie Z. Rahman, Babak Eliassi-Rad

**Affiliations:** 1 Department of Ophthalmology, Boston University, Boston, MA, USA; 2 Department of Ophthalmology, University of Texas Health, San Antonio, TX, USA

**Keywords:** Endocyclophotocoagulation (ECP), Phacoemulsification, African Continental Ancestry Group, Glaucoma, Primary Open-Angle Glaucoma, Chronic Angle-Closure Glaucoma

## Abstract

The aim of this study was to evaluate the outcomes of combined endocyclophotocoagulation (ECP) and phacoemulsification regarding vision, refraction, intraocular pressure (IOP), medication dependence, and complications in patients of African descent. A retrospective chart review was performed including all cases of ECP combined with phacoemulsification from October 2015 to March 2017. Exclusion criteria consisted of patients who were not of African descent and patients with <1 month follow-up. IOP was the primary outcome. Thirty-two eyes of 29 patients were included in the study. Mean ± standard deviation (SD) of IOP decreased from 17.30 ± 6.30 mmHg preoperatively to 15.88 ± 4.23 mmHg at the last postoperative visit (P = 0.301). In 2 of eight patients who did not demonstrate a difference in pre- and postoperative IOP changes, all IOP lowering medications were stopped. The mean ± SD of follow-up was 5.05 ± 4.08 months with a range of 1 to 18 months. The mean ± SD number of medications used for each patient for IOP control decreased from 2.59 ± 1.01 preoperatively to 1.97 ±1.38 (P = 0.045). Average visual acuity improved from 20/50 preoperatively to 20/25 (P = 0.002). Postoperative complication rate was low. ECP combined with phacoemulsification was effective to decrease IOP lowering medication dependence in patients of African descent. We found that combined ECP and phacoemulsification can lead to a significantly decreased dependence on IOP lowering drops, with some patients demonstrating complete independence from drops following surgery. Although there was not a significant decrease in IOP postoperatively when analyzed collectively, larger studies might to find such an association. Combined ECP and phacoemulsification has been shown to be a safe combination in patients with refractive glaucoma and may be considered if a patient desire less dependence on IOP lowering drops once other first-line methods have failed, or as a bridge between conservative and more definitive surgical treatment.

## INTRODUCTION

Minimally invasive glaucoma surgeries (MIGS) have risen in popularity in the recent years due to a higher safety profile than traditional, invasive glaucoma surgeries such as trabeculectomies or glaucoma drainage devices [1, 2]. Endocyclophotocoagulation (ECP) is a MIGS option used since 1980s and has commonly been reserved for refractory or end-stage glaucoma [3]. In such cases, all other treatment options including maximal medical therapy, laser and surgical intervention have already been used yet the patient’s glaucoma continues to progress or intraocular pressure (IOP) continues to remain above the acceptable standards. ECP involves the use of an endoscopic probe with a camera and diode laser to ablate the ciliary body. This results in decreased aqueous production and subsequently a lower IOP [4]. ECP has been shown to be successful as both a stand-alone procedure or in combination with cataract surgery [5]. Several studies have been published to assess the outcomes of ECP in advanced and refractory glaucomas including a prospective trial by Lima et al. [6] which compared ECP to the Ahmed glaucoma valve (AGV) in refractory glaucomas that have already undergone at least one prior trabeculectomy; they found no difference in the success rate between AGV and ECP. In fact, they found more complications by using AGV compared to the ECP. In another study, Kahook et al. [7] compared ECP treatment at one site versus two sites, evaluating the effect that extended treatment would have on IOP lowering. ECP has been reviewed in pediatric patients and also in studies looking for refractive outcomes [8, 9]. Recently, Smith et al. studied the effects of combined phacoemulsification and 360 degree ECP over 3 years in 84 patients with uncontrolled glaucoma and no prior history of glaucoma surgery. They found that 58.3% of cases had met failure criteria at the end of 3 years [10]. Despite the literature on ECP, there has only been one study comparing ECP outcomes in patients of African descent [11]. Given that refractory glaucoma is very common in this population compared to other populations, additional treatment options in these patients might be useful [12].

This retrospective study aimed to evaluate the outcomes of one-site ECP combined with cataract surgery for vision, IOP, refractive error and IOP lowering medication dependence to evaluate its effectiveness as an additional treatment option for patients of African descent.

## METHODS

The study followed the tenets of the Declaration of Helsinki, met local Ethical Clearance and was approved by the institutional review board (IRB) of Boston Medical Center. A retrospective chart review was performed of all cases of ECP (Beaver Visitek® Endo Optiks®, Waltham, MA, USA) combined with phacoemulsification cataract surgery via the Centurion® (Alcon, Fort Worth, TX) at Boston Medical Center from October 2015 to March 2017. Demographic data including age, race and gender were collected (Tables 1 and 2). Preoperative ocular parameters included glaucoma type and stage, previous surgeries, corrected distance visual acuity (CDVA), IOP, number of glaucoma medications used and surgical refractive target. Postoperative measures included CDVA, refraction, IOP (Table 1) and surgical complications. Glaucoma was staged according to the consolidated staging scheme developed by Ng et al. [13]. All types and stages of glaucoma were included in this study. Exclusion criteria included patients who were not of African descent and those who were lost to follow-up prior to one month. A paired t-test was used for statistical analysis of visual acuity, number of IOP lowering medications the patient remained dependent on and IOP at the latest visit.

**Table 1:** Demographics and Ocular Parameters

		P- value
Mean Age (Y)	68.4	
Total eyes (n)	32	
Gender		
**Female (n)**	20	
**Male (n)**	9	
Pre-op CDVA, (logMAR)	0.403 ± 0.454	
Post-op CDVA, (logMAR)	0.114 ± 0.226	**0.004**
Number of Glaucoma Medications pre-op (Mean ± SD)	2.59 ± 1.01	
Number of Glaucoma Medications in last visit (Mean ± SD)	1.97 ± 1.38	**0.004**
Pre-op IOP (Mean ± SD)	17.30 ± 6.30	
Post-op IOP, at last visit (Mean ± SD)	15.88 ± 4.23	
Glaucoma Type		0.130
**Advanced POAG, n (%)**	10/32 (31)	
**Moderate POAG, n (%)**	7/32 (22)	
**Mild POAG, n (%)**	3/32 (9.4)	
Indeterminate POAG*, n (%)	2/32 (6)	
**Advanced CACG, n (%)**	7/32 (22)	
**Advanced Uveitic glaucoma, n (%)**	1/32 (3)	
**Moderate Pigment Dispersion Glaucoma, n (%)**	1/32 (3)	
**Ocular Hypertension, n (%)**	1/32 (3)	
Previous Interventions		
**SLT, n (%)**	10/32 (31)	
**LPI, n (%)**	7/32 (22)	
**Trabeculectomy, n (%)**	2/32 (6)	
**Ahmed glaucoma valve, n (%)**	4/32 (12.5)	
**PRP, n (%)**	1/32 (3)	
**Pars Plana Vitrectomy, n (%)**	1/32 (3)	

Abbreviations: Y: year; n: number; CDVA: corrected distance visual acuity; F; female; M; male; SD: standard deviation; logMAR: Logarithm of the Minimum Angle of Resolution; IOP: intraocular pressure; %: percentage; POAG: primary open angle glaucoma; CACG: chronic angle closure glaucoma; SLT: selective laser trabeculoplasty; LPI: Laser Peripheral Iridotomy; PRP: Panretinal Photocoagulation: Pre-op: preoperative; Post-op: post operative. P<0.05 has been bold.

IOP pressure was measured using the Goldmann applanation (Haag-Streit Diagnostics, Bern, Switzerland) by a physician on 1 day, 1 week and 1 month following surgery for all patients. If patients were followed between the above listed visits or for longer than 1 month, the IOP at each visit and the latest IOP were recorded as well. Corrected distance visual acuity with Snellen chart and manifest refraction were completed at least 1-month postoperatively. Residual refractive outcomes were also evaluated by determining the difference between the spherical equivalent of the refractive goal and the postoperative manifest refraction. Statistical analysis was performed using Student’s 2-tailed T test on GraphPad Prism 6.0 (GraphPad Software Inc., San Diego, CA, USA).

**Table 2:** Ethnicity in Combined Endocyclophotocoagulation and Phacoemulsification in Patients with Glaucoma

Ethnicity	Number of eyes (Percentage)
African American	12 (41)
Haitian	11 (38)
Jamaican	2 (6)
Cape Verdean	2 (6)
Trinidadian	1 (3)
Moroccan	1 (3)

**Outcome Measures**

The primary outcome measured was IOP at the last visit. Secondary outcomes included CDVA at least 1 month after surgery, refractive outcomes, number of glaucoma medications and complications.

Complete success was defined as an IOP lowering of ≥ 20% from the preoperative amount while the patient remained off all glaucoma medications. Also, qualified success was defined as an IOP lowering of ≥ 20% while remaining on glaucoma medication(s).

**Surgical Method**

**Figure 1:**
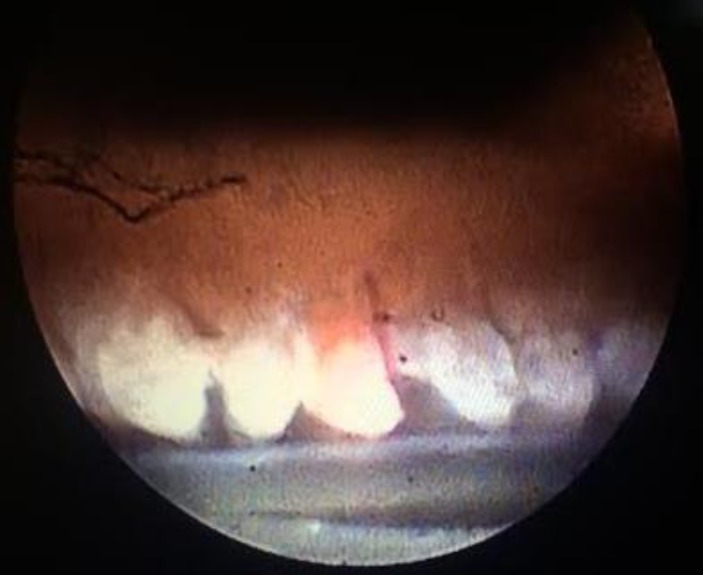
Intraoperative View of Ciliary Processes after Laser Ablation Following Endocyclophotocoagulation (ECP)

After phacoemulsification with intraocular lens (IOL) placement in the lens bag, the sulcus was inflated with viscoelastic (Provisc®, Alcon Ft. Worth, TX, The USA). A 19-gauge ECP (Beaver Visitek® Endo Optiks®, Waltham, MA, USA) probe was then inserted into the main wound and the camera was focused on the ciliary body processes with an optimal view of 4-6 ciliary processes at a time (Figure 1). A power of 0.20-0.25 milliwatt (mw) on a continuous setting was used to treat 4-6 clock hours (120 to 180 degrees) of ciliary body processes. The viscoelastic was then removed via the irrigation and aspiration handpiece and the wounds were sealed with balanced with salt solution.

**Postoperative Regimen and Follow-up**

The postoperative regimen included subconjunctival injection of cefazolin and dexamethasone and topical prednisolone acetate 1% (Allergan, Madison, NJ, The USA) 6 times per day tapered over 6 weeks, a fourth generation fluoroquinolone (Vigamox, Alcon, Ft. Worth, TX, The USA) 4 times a day for 1 week and ketorolac 0.5% (Allergan, Madison, NJ, The USA) 4 times a day for 1 month to the operated eye. Prostaglandin analogs were stopped immediately postoperatively; however, all other glaucoma medications were continued in the immediate postoperative period at day 0. The patient was followed at day 1, week 1 and month 1 and then as directed by the surgeon based on IOP and inflammation. IOP medications were then either added or subtracted at each subsequent visit at the surgeon’s discretion.

## RESULTS

**Demographics and Ocular Parameters**

Thirty-two eyes of 29 patients were included in the study. Twenty females and 9 males were included with a mean ± standard deviation (SD) age of 68.4 ±8.6 years (Table 1). Most patients were either of African American or Haitian decent but patients of Jamaican, Cape Verdean, Moroccan and Trinidadian descent were also included (Table 2).

**Table 3:** Mean Intraocular Pressure at Different Time Points in Combined Endocyclophotocoagulation and Phacoemulsification in Patients with Glaucoma of African Descent

Time Period	IOP	Number of eyes
Day 1	21.4 ± 8.0	32
Week 1	17.3 ± 4.9	32
Month 1	17.7 ± 4.5	32
IOP Last		
**1-3 Months**	16.4 ± 2.0	13
**4-6 Months**	15.1 ± 3.8	12
**7-10 Months**	14.7 ± 2.3	3
**11-18 Months**	19.5 ± 3.9	4

*IOP measured in millimeter of mercury (mmHg)

Data in table are presented as Mean ± SD (Standatd Deviation)

Ten of 32 (31%) eyes had advanced primary open angle glaucoma (POAG), 7 (22%) had moderate POAG, 3 (9%) mild POAG and 2 (6%) an indeterminate stage of POAG. Seven of 32 (22%) had advanced chronic angle closure glaucoma (CACG). There was one patient each with ocular hypertension, uveitic glaucoma and pigment dispersion glaucoma. Previous laser intervention and surgical treatment are represented in Table 1.

**IOP and Medications**

**Figure 2:**
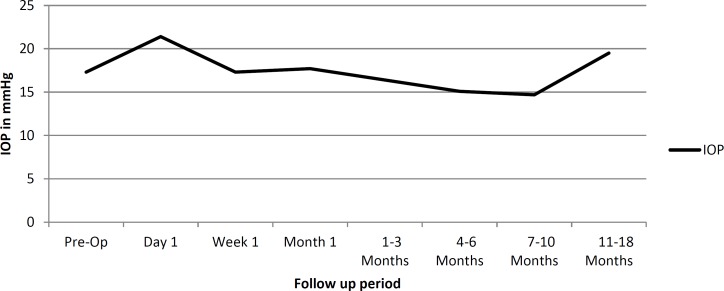
Intraocular Pressure (IOP) in Millimeters of Mercury (mmHg) over Time

The pre- and postoperative IOP and number of glaucoma medications used pre- and postoperatively are listed in Tables 1 and 3. The mean ± SD of preoperative IOP was 17.3 ± 6.3 mmHg. The postop IOP was reviewed for each patient on postoperative day 1, week 1 and month 1 as well as on the latest visit after one-month postoperative period. The mean ± SD of patient follow-up was 5.05 ± 4.08 months with a range of 1 to 18 months. At day 1, the mean ± SD of IOP was 21.4 ± 8.0 mmHg, at week one 17.3 ±4.9 mmHg and at month one 17.7 ± 4.5 mmHg. Thirteen eyes followed up 3 months after surgery and the mean ± SD of IOP for this time frame was 16.4 ± 2.0 mmHg. Twelve eyes completed the follow-up between 4 and 6 months after surgery and the mean ± SD of IOP was 15.1 ± 3.8 mmHg. Three eyes had follow-up between 7 and 10 months and the mean ± SD of IOP in this group was 14.7 ±2.3 mmHg. Four eyes had follow-up between 11 and 18 months after surgery and the mean ± SD of IOP was 19.5 ±3.9 mmHg (Table 3, Figure 2). The mean ± SD of IOP at the last visit for all patients without considering length of follow-up after one month postoperatively was 15.88 ±4.23 mmHg compared to 17.3 ±6.30 mmHg preoperatively (p = 0.130). This represented an 8.2% drop in IOP compared to before surgery. Two of 32 patients (6%) achieved complete success while 9 (28%) achieved a qualified success. Also, 6 patients (67%) in the qualified success group were able to achieve an IOP lowering of >30% and two patients an IOP lowering of >40%. Eight of 32 patients (25%) ended up with higher IOPs than their preoperative measures. Additionally, 7 (22%) experienced no change in IOP compared to their preoperative measures. However, 2 of these 7 patients were able to be weaned off of their IOP lowering medications completely. In the qualified success group, 6/9 (66%) had moderate to advanced POAG. Of the 8 that worsened, 6 had POAG and the other 2 had CACG.

 Preoperatively, the mean ± SD of number of glaucoma medications was 2.59 ±1.01. At last visit after surgery, the mean ± SD of number of glaucoma medications was 1.97 ±4.23 showing a 24% reduction (P = 0.004).

**Visual Acuity and Refractive Outcomes**

The mean± SD of CDVA in logMAR were 0.57±0.68 and 0.15±0.26 preoperatively and at last visit postoperatively, respectively (p = 0.002) as shown in Table 1. Refractive outcomes were also evaluated by comparing postoperative spherical equivalent to the spherical equivalent of the refractive goal based on the IOL calculations obtained preoperatively. A target refraction within 1.0 diopters (D) of the refractive target was considered meeting the patient’s refractive goal as described by Simon et al. [14]. Twenty-seven of 32 eyes met the refractive goal. However, 5 of 32 eyes (16%) were >1.0D away from their goal, whereby 3 of these eyes experiencing a myopic surprise and 2 a hyperopic surprise. Additionally, 14 of 32 eyes (44%) were found to be > ± 0.50D of goal with 10 of these eyes ending up more myopic and 6 of these eyes ending up more hyperopic. Overall, 18 of 32 eyes (56%) were found to be within ± 0.50D of goal (Table 4).

**Complications**

Complications were noted as early (less than 1 month after surgery) or late (greater than 1 month after surgery) and occurred in 14 of 32 eyes (Table 5). The most common complication was an IOP spike defined as an increase in IOP of > 10mmHg from baseline on postoperative day 1 (POD 1) which occurred in 8 of 32 eyes (25%). These patients were treated with topical IOP lowering medications and IOP was stabilized prior to the patient leaving clinic. Cystoid macular edema (CME) occurred in 2 of 32 eyes (6%). Prolonged postoperative inflammation occurred in 1 of 32 eyes (3%) but resolved with topical steroids. One patient developed a hyphema on POD 1 which resolved by the following week. Another patient developed a steroid response that required an additional Baerveldt glaucoma implant to adequately control the IOP.

**Table 4:** Refractive Outcomes of Combined Endocyclophotocoagulation and Phacoemulsification in Patients with Glaucoma of African Descent

Outcome	n (%)	Mean ± SD
>±1.0 D of goal	5/32 (16)	
Myopic surprise	3/5	-1.12 ± 0.15
Hyperopic surprise	2/5	+1.27 ± 0.14
>±0.50 D of goal	14/32 (44)	
Myopic deviation	8	-0.78 ± 0.25
Hyperopic deviation	6	+0.91 ± 0.30
Within ± 0.50D of Goal	18/32 (56)	+0.02 ± 0.30

Abbreviations: D: diopter; SD: standard deviation; %: percentage; n: number of eyes.

**Table 5:** Complications Combined Endocyclophotocoagulation and Phacoemulsification in Patients with Glaucoma of African Descent

Early Complications (< 1 month)	**n (%)**
Postoperative day 1 IOP Spike	8/32 (25)
**Post-operative Iritis**	1/32 (3)
**Hyphema**	1/32 (3)
Late Complications (> 1 month)	
**Cystoid Macular Edema**	2/32 (6)
**Steroid Response**	1/32 (3)
**Further Surgery (Baerveldt glaucoma implant)**	1/32 (3)

Abbreviations: IOP: Intraocular pressure; SD: standard deviation; mmHg: millimeters of Mercury ; %: percentage; n: number.

## DISCUSSION

Our study aimed to look at ECP combined with phacoemulsification outcomes specifically in patients of African descent with varied types and stages of glaucoma. We demonstrated that ECP combined with phacoemulsification can be effective to decrease IOP and/or lowering medication dependence in patients of African descent. We also found that combined ECP and phacoemulsification is safe with no vision threatening complications. The most common complication was elevated IOP on postop day 1 visit (8 of 32 patients, 25%).ECP has been used since the 1980s for the treatment of many types of glaucoma [3]. Francis et al. [15] showed that ECP combined with phacoemulsification was more effective than cataract extraction alone to lower IOP and decrease IOP lowering medication dependence specifically in eyes with mild to moderate glaucoma previously controlled with IOP lowering medications. However, a recent study by Smith et al. indicated that these results are temporary [10]. Lima et al. [6] in a prospective study comparing ECP to AGV in patients with refractory glaucoma who were pseudophakic and had undergone trabeculectomy with antimetabolite previously, demonstrated that IOP lowering was similar between the two groups and the ECP group had less complications comparted to the AGV group. Another prospective study by Gayton et al. [16] comparing ECP combined with phacoemulsification to phacoemulsification alone in patients with prior trabeculectomy found comparable IOP lowing results and decreased IOP lowering medication dependence. A retrospective review by Chen et al. also found that ECP is safe and effective in refractory glaucomas of various types, including in patients who have had previous trabeculectomy or tube shunts [17]. Recently, Murakami et al. noted that ECP functions equivalently to a second glaucoma drainage device in eyes that have had a prior implanted drainage device [18]. ECP has even been studied in difficult pediatric glaucoma including congenital glaucoma, aphakia, Sturge-Weber syndrome and aniridia [8, 19]. Despite the limited but varied data regarding ECP, only one previous study has looked specifically at results of ECP in a population of patients of African descent. This study found that postoperative inflammation is higher in patients of African descent but did not find any differences in IOP or best corrected visual acuity (BCVA) compared to patients of Caucasian decent [11]. Studying the effects of combined ECP procedures in patients of African descent is important since refractory glaucoma is very common amongst these individuals despite maximal medical therapy and surgical interventions, thus rendering further options necessary [11, 20].

In our retrospective study, it appears that four patients received a robust IOP lowering of up to 40%. Specifically, a patient with severe stage CACG post-trabeculectomy had a 42% reduction, another patient with severe stage POAG post-AGV demonstrated a 42% reduction, a patient with severe stage POAG post selective laser trabeculoplasty (SLT) had a 38% reduction and a patient with moderate POAG with no prior intervention showed a 38% reduction. However, some patients did not respond to combined phacoemulsification and ECP. When analyzing subsets of our population, 3 patients who had undergone either a prior trabeculectomy or AGV achieved a significant IOP lowering. Combined ECP and phacoemulsification may be quite effective in these particular patients, which is consistent with results of some prior studies [6, 17]. Combined ECP and phacoemulsification was successful in 6 of 9 (67%) patients who had POAG. In seven of thirty eyes (22%) IOP worsened following the procedure. Of these seven eyes, four of them were receiving fewer IOP lowering drops postoperatively than preoperatively. Latanoprost or an equivalent prostaglandin analog was stopped after surgery. Perhaps discontinuing IOP lowering medications postoperatively caused an increase in IOP. Additionally, 2/7 (29%) patients who had a higher postoperative IOP developed CME. One patient with CME had POAG and another had CACG. Of the patients who demonstrated worsening IOP postoperatively, there was a mix of glaucoma status and types and none had undergone prior filtering or tube shunt surgeries. In our study, 4-6 clock hours, or 120-180 degrees of ciliary processes were treated through a single incision. While a previous study by Uram [21] found the efficacy of the same amount of treatment, a few others suggested a greater treatment area to achieve adequate IOP lowering. One such study by Patel et al. recommended treating an area of at least 180 degrees while Zabrin et al. found treating 240 degrees to be more effective [3, 22]. More recently, Kahook compared 1-site of 240 degree ECP treatment versus 2-site ECP treatment of 240-360 degrees and found that 2-site ECP combined with phacoemulsification resulted in a statistically significant lowering of IOP compared to 1-site ECP treatment. He also noted that 2-site ECP treatment resulted in less IOP medication dependence. Besides, there was no difference in inflammation or complication rate in the 2-site group compared to the 1-site group. Our lower complication rate in comparison to other studies may be related to the relatively smaller degree of ciliary processes treated; however, larger treatment areas may be more effective and may have a similar safety profile compared to a smaller treatment area [7]. This may be a future area of comparison in patients of African descent.

The complication rate was low with a POD 1 IOP spike being the most common complication. This IOP spike resolved in all patients with the use of topical IOP lowering medications in the office. After administration of oral Diamox 500mg postoperatively to all patients, no further POD 1 IOP spikes were noted. Only one patient required an AGV to further lowering IOP; however, this patient had a preoperative IOP of 48 mmHg and post-ECP/phacoemulsification measure dropped to 28 mmHg. Combined ECP/phacoemulsification was attempted in this patient prior to an AGV since the patient requested a less invasive procedure prior to considering a drainage device. No patients developed severe complications such as hypotony, choroidal detachments or fibrin in the anterior chamber. As mentioned above our lower complication rate in comparison to other studies could be related to the relatively smaller degree of ciliary processes treated. Only one of 32 eyes (3%) developed a small hyphema which resolved without complication and one eye of another patient (3%) developed postoperative iritis which resolved with topical steroids. Both of these patients had POAG. In total, two of 32 eyes (6%) developed CME. Our complications rate is lower than that reported by Chen et al. [17]. For example, we had no cases of fibrin in the anterior chamber compared to 22% [17]. The hyphema rate (3%) was lower in our study compared to 12% reported by Chen et al. [17]. Our CME rate was 6% which is lower compared to 10% [17]. Our lower complication rate may be attributed to a smaller treatment area as noted previously.

Postoperatively, 2 of 32 eyes (6%) achieved complete success with an IOP lowering of ≥ 20% without consuming any IOP lowering medications. Despite the low percentage of complete success, no other studies used this strict criterion for complete success in the setting of ECP. Both patients with complete success were of Haitian decent and had POAG. Qualified success, defined as IOP lowering of 20% from baseline at the last visit regardless of IOP lowering medication use was found in 9 of 32 eyes (28%). Similar criteria for qualified success have been used in other studies in the glaucoma literature [23, 24]. In the qualified success group 5 were of Haitian decent. Seven of 9 had moderate to advanced POAG, 1 advanced CACG and 1 ocular hypertension. Thus, considering the complete and qualified success groups as a whole, POAG and Haitian descent associated with significant IOP lowering postoperatively. Compared to the literature, our complete and qualified success rates are lower than those found in a recent study by Clement et al. who used similar success criteria and found success in 55.5% of eyes at 12 months [24]. Perhaps this higher success rate was due to a larger area of treatment and shorter follow-up time. In our study, patients who were followed for greater than 12 months demonstrated a rise in IOP suggesting that the pressure lowering effect of ECP is only temporary. However, due to limited length of follow-up and only 4 of 29 patients completed a 12-month follow-up, it would be difficult to make that conclusion without a larger sample size. ECP results have been variable, with some studies showing continued IOP lowering success for up to 2-3 years [6, 10, 25]. Perhaps our lower success rate over time could be attributed to our population which has a higher proportion of advanced and refractory glaucoma, and to our smaller ECP treatment zone. Overall, IOP was decreased on average by 5% from baseline and all patients were able to be weaned off of one IOP lowering medication averagely. Roberts et al. found that combined ECP and phacoemulsification resulted in lower IOPs at early stages, but IOP steadily increased throughout the 12-month follow-up. This study stratified outcomes based on race but did not find any statistical difference amongst race. It noted that the only outcome associated with successful treatment was preoperative IOP [25]. Our results are similar to a recent review of ECP combined with phacoemulsification by Rathi and Radcliffe [26]. Although we did not find a statistically significant drop in IOP status post-treatment, we did find a statistically significant decreased dependence on IOP lowering medication following treatment.

CDVA improved after surgery, the outcome was excellent with an average CDVA of logMAR 0.15 (Snellen acuity of 20/28), including patients with limited visual potential due to advanced glaucoma or retinal pathology (i.e. epiretinal membrane). Furthermore 19/32 (59%) ended up with a CDVA of 20/20 (logMAR 0) and 28/32 (88%) achieved a BCVA of >20/30. Such visual acuity results is comparable to a study by Siegel et al. [27] which compared the outcome of ECP and phacoemulsification with phacoemulsification alone. This is the third investigation in the literature to our knowledge to assess refractive outcomes. In a retrospective case series, Wang et al. [28] compared refractive results of combined ECP and phacoemulsification to phacoemulsification alone. They found that in combined ECP, refractive outcomes were less predictable and patients often experienced a small myopic shift. Moreover, another retrospective case series by Kang et al. [8] found more refractive variability postoperatively in eyes who underwent ECP combined with phacoemulsification compared to those who underwent phacoemulsification alone. We found 84% of patients to be within ±1.00 D which is similar to Kang et al. finding of 90% to be within this goal. Furthermore, only 48% of patients with combined ECP were within ±0.50D of goal, while 100% of phacoemulsification without ECP cases were within ±0.50 D of goal [9]. In our study, 5/32 (16%) were > ±1.0 D of goal. Of these 5 cases, 3 resulted in a myopic surprise and 2 a hyperopic surprise. Four of 5 of these patients, 3 with POAG and 1 with CACG, who obtained a refractive surprise had advanced glaucoma, suggesting that patients with advanced disease are more likely to experience a refractive surprise probably. Sixteen of 32 eyes (56%) ended up within ± 0.50 D of goal. These outcomes suggest that physicians should take extra care to adjust for IOL power. Currently no formula exists to adjust for ECP combined phacoemulsification refractive outcomes. The quality of each preoperative biometry, A-scan, and Keratometry readings were reviewed in our study and found to be accurate. One explanation for the variable refractive surprises may be that the lens/zonule complex is attached to the ciliary body, which is non-uniformly ablated during ECP. Moreover, ablating only a small section of ciliary bodies could result in shifts of the entire lens complex. Considering our study and the 2 prior studies, postoperative refractive surprises may be possible and patients need to be counseled accordingly.

The strength of our study is the patient demographics. Our study population included subjects of African descent, as glaucoma is more common. Glaucoma is a leading cause of decreased vision and/or blindness in this population, hence it is important to analyze different surgical options such as ECP combined with phacoemulsification in this group. Additionally, we included all types and stages of glaucoma in our study, so that we could assess whether there is a certain subgroup in which ECP combined with phacoemulsification is more successful and safe. The limitations of our study were the small sample size, the design of the study (retrospective case series) and the short duration of study (mean follow-up of 5 months, with a range of 1-18 months). We suggest more studies with larger sample size, longer follow-up period and more valid study design to achieve more robust conclusion.

## CONCLUSIONS

Combined phacoemulsification and ECP had a low complication rate and may serve as a helpful and safe procedure in the management of glaucoma. In particular, combined ECP/phacoemulsification may work better in patients with POAG who have had prior glaucoma surgery when further surgical options are limited. Due to the variability in refractive outcomes after surgery, preoperative planning should be considered. Limitations include the retrospective nature of our study, short follow-up period with only 4/29 patients who followed up to 12 months, lack of a control group and a small sample size. In addition, a larger treatment area in addition to 4-6 clock hours might result in a higher success rate than what was reported. Overall, ECP combined with cataract surgery can achieve robust IOP lowering in some patients while achieving no improvement or even worsening IOP in some patients of African descent. In patients who did not show improvement in IOP following surgery, relying on standard surgical interventions such as AGV or trabeculectomy may be necessary. Despite variability in IOP outcomes, there was a significant decrease in reliance on IOP lowering medications in all patients. Finally, combined phacoemulsification and ECP may serve as an additional treatment measure in patients with moderate/advanced POAG who have already undergone trabeculectomies or AGV and still need additional intervention. Combined phacoemulsification and ECP may also serve as a bridge in patients who have received maximum medical therapy but not interested in undergoing an invasive glaucoma surgery.

## DISCLOSURE

Ethical issues have been completely observed by the authors. All named authors meet the International Committee of Medical Journal Editors (ICMJE) criteria for authorship of this manuscript, take responsibility for the integrity of the work as a whole, and have given final approval for the version to be published. No conflict of interest has been presented.

## Funding/Support

None.
